# The pterygopalatine fossa: imaging anatomy, communications, and pathology revisited

**DOI:** 10.1007/s13244-016-0498-1

**Published:** 2016-05-26

**Authors:** Sonam Tashi, Bela S. Purohit, Minerva Becker, Pravin Mundada

**Affiliations:** Department of Diagnostic Radiology, Changi General Hospital, 2 Simei Street 3, Singapore, 529889 Singapore; Department of Neuroradiology, National Neuroscience Institute, 11 Jalan Tan Tock Seng, Singapore, 308433 Singapore; Department of Imaging, Division of Radiology, Geneva University Hospital, Rue Gabrielle Perret Gentil 4, 1211 Geneva 14, Switzerland

**Keywords:** Pterygopalatine fossa, CT, MRI, Perineural tumour spread

## Abstract

**Abstract:**

The pterygopalatine fossa (PPF) is a small, clinically inaccessible, fat-filled space located in the deep face that serves as a major neurovascular crossroad between the oral cavity, nasal cavity, nasopharynx, orbit, masticator space, and the middle cranial fossa. Due to its inherent complex location and connections, it can potentially act as a natural conduit for the spread of inflammatory and neoplastic diseases across the various deep spaces in the head and neck. This review aims to acquaint the reader with the imaging anatomy of the PPF, its important communications, and to identify some major pathological conditions that can involve the PPF, especially in conditions where its involvement can have serious diagnostic and therapeutic implications, such as in perineural tumour spread.

***Teaching points*:**

• *The PPF is a small neurovascular junction in the deep face with important to-and-fro connections.*

• *Awareness of anatomy of the PPF and its communications helps to simplify imaging of its pathology.*

• *Perineural tumour spread is clinically the most important pathology in this region.*

## Introduction

The pterygopalatine fossa (PPF) is an obscure but important space in the deep face that needs to be evaluated very carefully in the realm of head and neck imaging, since a myriad of infective, inflammatory, and neoplastic conditions can affect it. These conditions may not only be difficult to detect clinically, but also difficult to treat, if not diagnosed accurately. The multiple communications of the PPF with the deep neck spaces serve as ready pathways for loco-regional spread of disease. Therefore, familiarity with its complex anatomy and thorough understanding of the imaging features of its common pathologies are mandatory to improve diagnostic accuracy as well as patient management [1–7].

## Imaging anatomy, contents, and major communications of the PPF

**Fig. 1:**
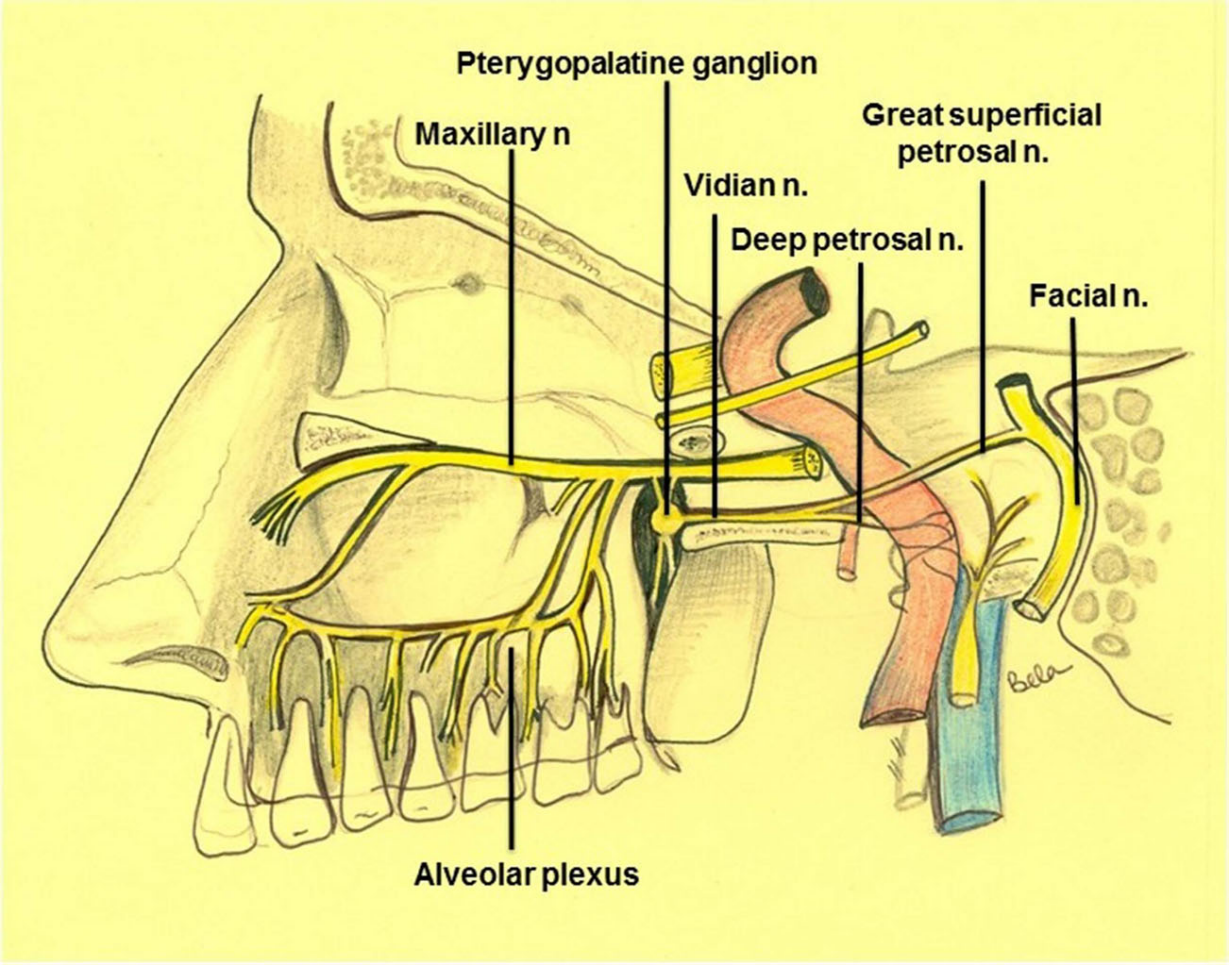
Schematic diagram showing the bony anatomy and main neural connections of the PPF. Illustration by Dr. Bela Purohit

The PPF is an inverted pyramid-shaped space bounded by the junction of the maxilla, palatine, and sphenoid bones. The PPF contains fat, the pterygopalatine ganglion (PPG), the maxillary division (V2) of the trigeminal nerve and its branches [zygomatic nerve, posterior superior alveolar nerve(s), and the infraorbital nerve (ION)], the Vidian (pterygoid) nerve, the distal branches of the maxillary artery, and a few emissary veins [1–7] (Fig. 1).

High-resolution computed tomography (HRCT) is the modality of choice for evaluating the various bony communications of the PPF. Typically, very thin axial sections are obtained on a multidetector CT scanner, parallel to the lower borders of the upper teeth, starting from the hard-palate to the mid-hypophyseal fossa. Coronal reformats are obtained parallel to the posterior wall of the maxillary sinus back to the level of the posterior clinoids [4].

**Fig. 2:**
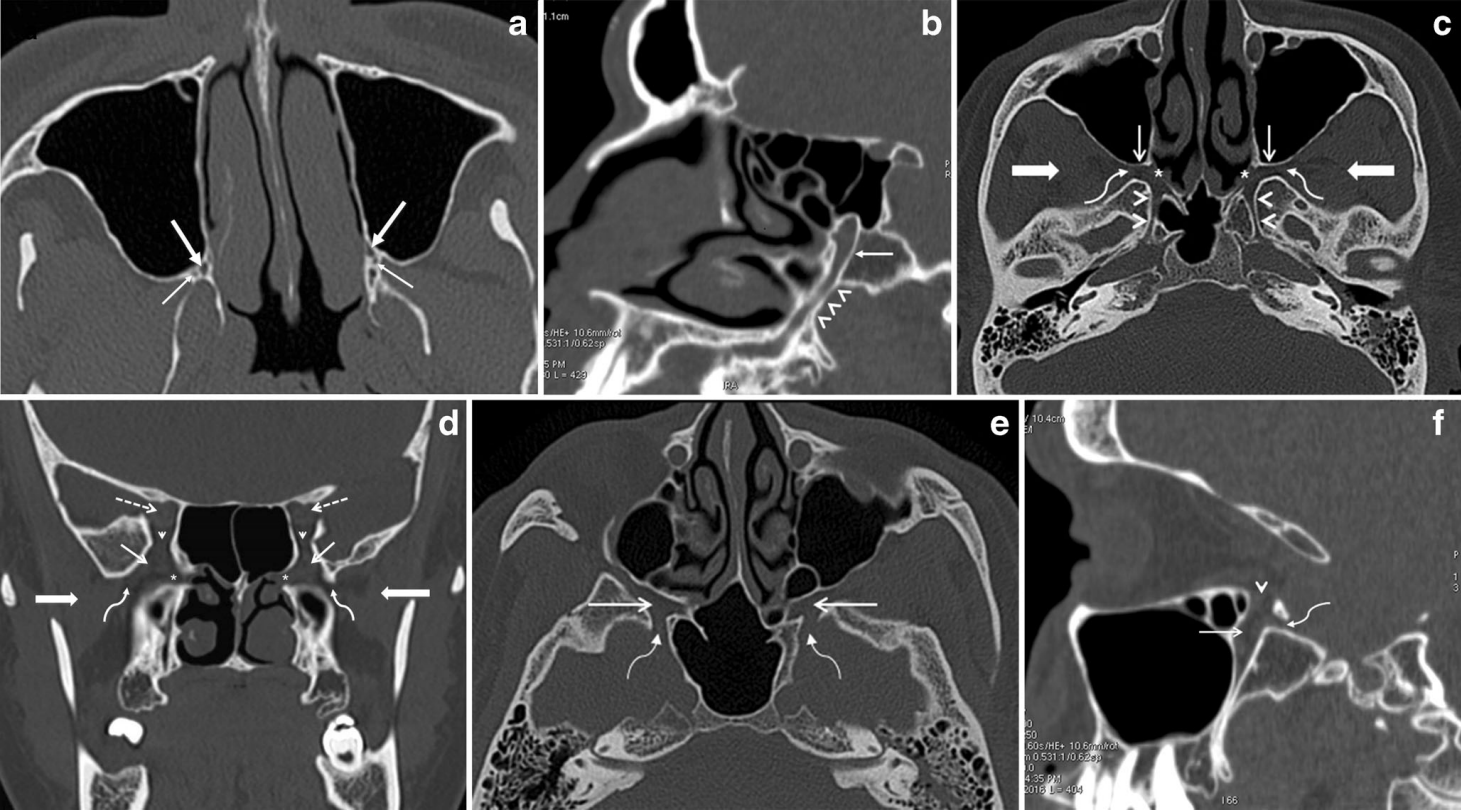
**a** Axial HRCT image shows the greater (*thick arrows*) and lesser (*thin arrows*) palatine canals at the inferior apex of the PPF. **b** Oblique HRCT reconstruction through the greater palatine canal (*arrowheads*) shows its entire longitudinal course, from the hard palate inferiorly to the apex of the PPF superiorly (*arrow*). **c** Axial HRCT image through the PPF obtained at the level of the Vidian canal. The PPF (*thin straight arrows*) communicates medially with the nasal cavity via the SPF (*asterisk*) and laterally with the ITF (*thick straight arrows*) via the PMF (*curved arrows*). The Vidian canal (*arrowheads*) extends from the PPF to the foramen lacerum on each side. **d** Coronal HRCT image through the PPF at the level of the IOF. The PPF (*thin straight arrows*) communicates with the ITF (*thick straight arrows*) via the PMF (*curved arrows*). The SPF (*asterisk*) opens into the nasal cavity medially and the IOF (*arrowheads*) opens into the orbital apex (*dashed arrows*). **e** Axial HRCT image through the PPF obtained at the level of the foramen rotundum. The PPF (*straight arrows*) communicates with the middle cranial fossa via the foramen rotundum (*curved arrow*) on each side. **f** Sagittal HRCT reconstruction through the PPF (*straight arrow*) showing its communication with the orbit via the IOF (*arrowhead*) and with the middle cranial fossa via the foramen rotundum (*curved arrow*) at the same level

By using the aforementioned planes of acquisition, the most caudal structure of the PPF (apex of pyramid) as identified on axial HRCT sections, is the greater palatine canal, which descends within the posteroinferior aspect of the medial wall of the maxillary bone to open as the greater palatine foramen in the posterolateral aspect of the hard palate (Fig. 2a and b). The lesser palatine canal is seen posterior to the greater palatine canal, traversing the pyramidal process of the palatine bone and opening as the lesser palatine foramen at its inferior aspect. The lesser and greater palatine canals house the lesser and greater palatine nerves, respectively; these serve as efferent branches from the PPG to the palate. Upon scrolling up, the PPF appears as a small oval space bounded anteriorly by the posterior wall of the maxillary sinus, medially by the palatine bone and posteriorly by the pterygoid plates (Fig. 2b). Laterally, the PPF communicates with the infratemporal fossa (ITF) via the pterygomaxillary fissure (PMF) (Fig. 2c and d). The roof of the PPF is formed by the sphenoid bone. The PPF communicates with the foramen lacerum via the Vidian canal (Fig. 2c), which traverses posterolaterally through the body of the sphenoid bone. The Vidian canal contains the Vidian nerve, which carries parasympathatic preganglionic fibres from the facial nerve to the PPG. The postganglionic fibres from the PPG supply the lacrimal gland and the mucosa of the nasal cavity and nasopharynx. At about the same level, the PPF also communicates with the nasal cavity via the sphenopalatine foramen (SPF) (Fig. 2c and d), just anterior to the Vidian canal opening. At its most cranial aspect (base of pyramid), the PPF communicates with the orbit via the inferior orbital fissure (IOF) (Fig. 2d and f). The IOF transmits the zygomatic branch of V2, ascending branches from the PPG and the ION. At the same level, the foramen rotundum (Fig. 2e and f) also enters the posterosuperior aspect of the PPF, communicating with the middle cranial fossa. The V2 nerve travels through the foramen rotundum to enter the PPF and continues further as the ION [1–4].

While CT is useful for studying bony anatomy, magnetic resonance imaging (MRI) is excellent for evaluating pathologies of the PPF, especially perineural tumour spread (PNS). High-resolution, thin-section (2–3 mm), small field-of-view MRI using a head-coil is recommended for evaluating the PPF and its connections. Precontrast axial SE T1W, axial FSE T2W and coronal SE T1W images followed by gadolinium-enhanced, fat-saturated (FS) axial and coronal SE T1W images are routinely acquired [5–7]. 3D FT T1W sequences with 0.6–1-mm-thin slices (VIBE-Volumetric Interpolated Examination) after intravenous contrast administration are additionally used by some investigators including ourselves. These volumetric data sets allow multiplanar reconstructions in any given plane and thereby facilitate the evaluation of subtle abnormalities [8].

## Key CT and MR imaging appearances in PPF pathology

**Fig. 3:**
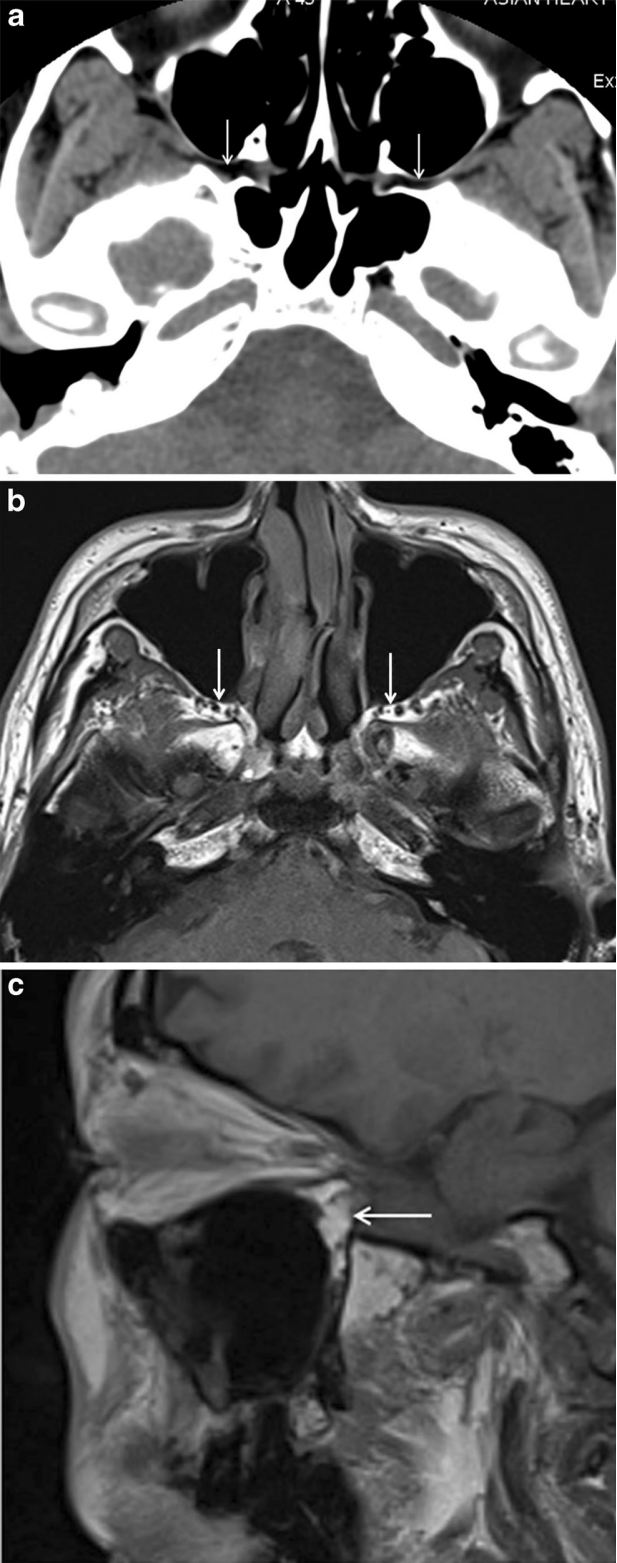
**a** Axial NECT image (soft-tissue window) showing normal hypodense appearance of bilateral fat-filled PPF (*arrows*). **b** and **c** Axial T1W MR image and sagittal T1W MR image showing hyperintense appearance of normal fat-filled PPF (*arrows*)

On non-contrast enhanced CT (NECT) with soft-tissue window settings, the normal PPF appears as a hypodense, fat-density space (Fig. 3a). On T1W MR images, normal fat within the PPF shows hyperintense signal (Fig. 3b and c). It may contain small flow voids from branches of the internal maxillary artery. The PPF is not seen on imaging. Mild post-contrast MR enhancement within the PPF is normal, due to the presence of small emissary veins [1, 5, 7].

Obliteration or replacement of the fat with soft tissue, widening of the PPF, and bony erosion of its walls are key imaging findings of its pathological involvement. Enlargement of communicating neural foramina and/or infiltration of the fat within the neural foramina suggests PNS. Osseous abnormalities are best seen on CT whilst replacement of fat is best seen on T1W MR images. Contrast-enhanced T1W MR images, especially with fat-saturation, can elegantly demonstrate PNS within the PPF [5–8].

## PPF pathology

Various neoplastic as well as infective and inflammatory conditions are known to involve the PPF. These pathologies can extend to the PPF either by direct spread or via the neurovascular communications. The presence of disease like PNS within the PPF has serious treatment and prognostic implications—it often portends recurrence, need for wider surgical resection, expansion of radiotherapy (RT) portals, and at times, poor prognosis [5–7, 9].

## Neoplastic conditions

The PPF can be involved either by direct tumour invasion or by PNS. Direct invasion of the PPF is often seen in tumours of the nasal cavity/nasopharynx like juvenile nasopharyngeal angiofibroma (JNA), nasopharyngeal carcinoma (NPC), and in masticator space sarcomas. PNS is a well-known form of spread in head-neck cancers in which there is gross, radiologically evident disease spread along large nerves. It commonly occurs in a retrograde fashion, toward the central nervous system. Since the PPF is a central station in the trigeminal nerve pathway, it is often the site of PNS, typically from cancers of the palate, cheek, maxillary sinus, and nasopharynx [5–7, 9–11]. Imaging is critical for diagnosing PNS as it may be clinically silent in about 40 % of patients [6].

### Direct tumour invasion into the PPF

#### JNA

**Fig. 4:**
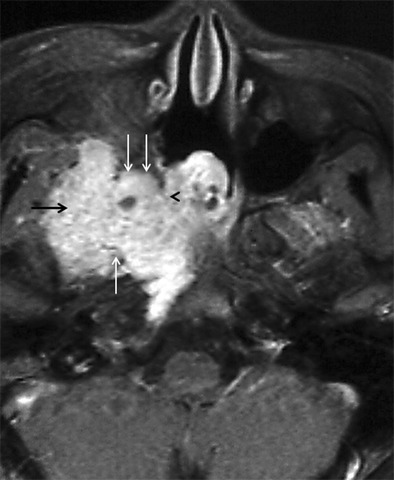
**a** JNA in a 17-year-old male who presented with recurrent epistaxis. Axial contrast-enhanced, FS T1W MR image shows a large, avidly enhancing mass in the right nasopharynx and PPF. The tumour extends from the right SPF (*arrowhead*) into the right PPF, expanding its walls (*thin white arrows*), and onwards laterally into the right ITF (*black arrow*)

JNA is a benign, vascular, locally aggressive tumour, which originates in the SPF. Its direct lateral extension into the PPF is considered the most important event in its expansion, as it can then have multidirectional invasion into the maxillary sinus anteriorly, the ITF laterally via the PMF (Fig. 4), the pterygoid fossa posteriorly, and the orbit/cranial fossa superiorly via the IOF. Besides the IOF, JNA can extend from the PPF further intracranially via the Vidian canal. Locally, it produces widening of the PPF with bowing and erosions of its bony walls. The multidirectional extension of JNA from the PPF is associated with higher morbidity due to the involvement of more important structures as opposed to its multidirectional extension from its origin in the SPF [10–13].

Over the years, various staging systems based on tumour extension have been utilized for stratifying patients with JNA. These systems identify lesions amenable to resection by either endoscopic, external, or combined approach, and also the extent of resection. Commonly used staging systems are from Sessions et al., Andrews et al., Radkowski et al., Onerci et al., and Carrillo et al. In most of these, involvement of the PPF is classified as stage II, whereas involvement of ITF or intracranial extension is classified as stage III or IV. Currently, endoscopic excision of JNA extending up to the PPF is considered as standard treatment with high rates of complete resection and minimal morbidity. However, lesions extending into the ITF or intracranially require external approaches, which are technically more challenging. Additional craniotomy and RT may be required for extensive disease [10–13].

#### NPC

**Fig. 5:**
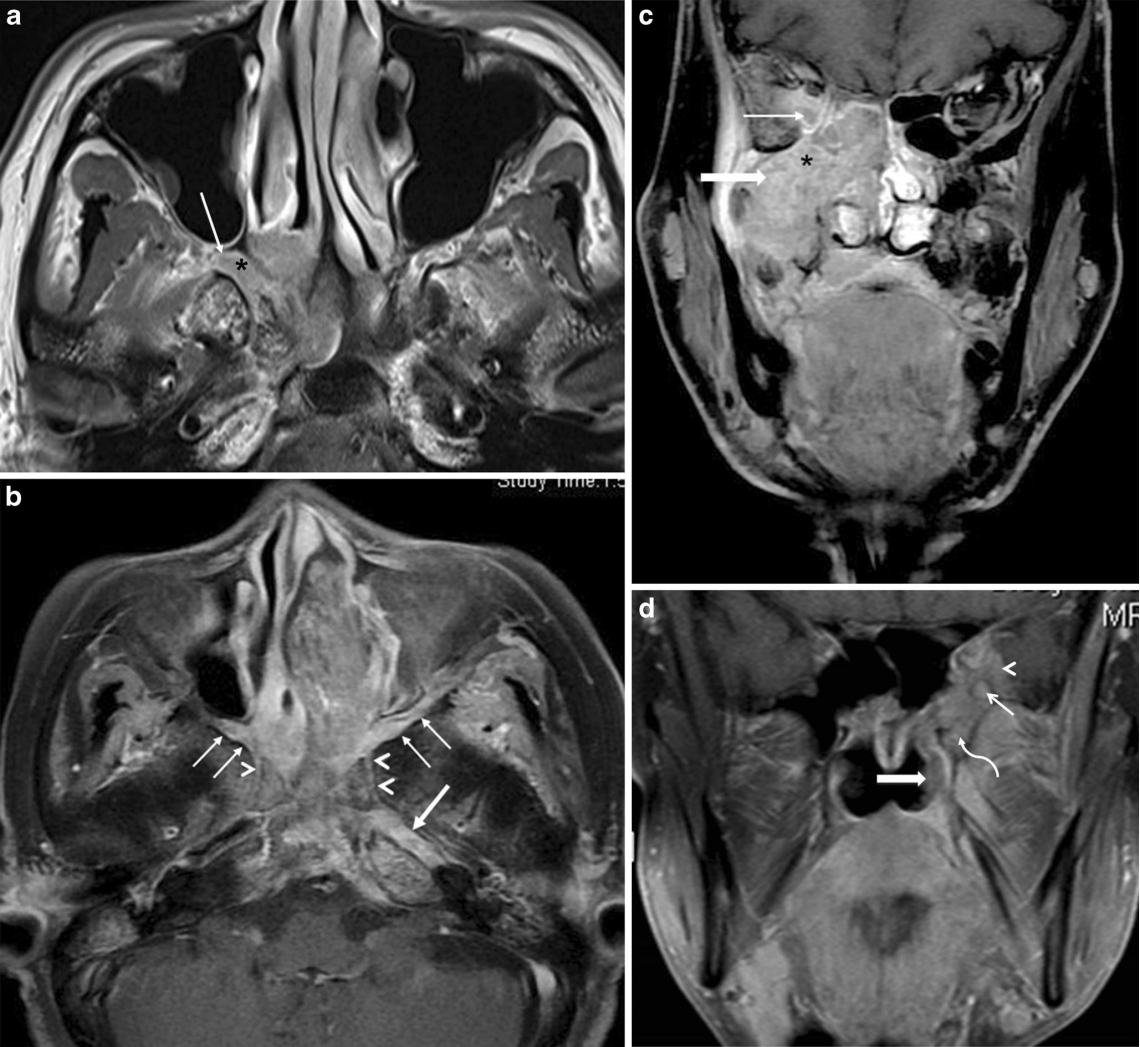
**a** NPC in a 50-year-old male patient. Axial contrast-enhanced T1W MR image shows an enhancing mass in the right side of the nasopharynx with direct extension into the medial half of the right PPF (*arrow*) via the right SPF (*asterisk*). **b** NPC in a 46-year-old male patient. Axial contrast-enhanced, FS T1W MR image shows an enhancing mass in the nasopharynx extending into the left nasal cavity and showing bilateral involvement of the PPFs (*thin arrows*). From the PPFs, there is retrograde PNS via bilateral Vidian nerves (*arrowheads*) with extension up to the left foramen lacerum (*thick arrow*). **c** Extensive stage 4 NPC in a 60-year-old male patient. Coronal contrast-enhanced FS T1W MR image shows extension of the tumour into the right ITF (*thick arrow*) and into the right orbit (*thin arrow*) via the PPF (*asterisk*). **d** Recurrent NPC in a 55-year-old female patient. Coronal contrast-enhanced FS T1W MR image shows an enhancing soft-tissue mass in the left side of the nasopharynx (*thick arrow*), with extension into the left PPF (*curved arrow*), leading to PNS along the left foramen rotundum (*thin straight arrow*). There is also involvement of the left middle cranial fossa (*arrowhead*)

NPC is an aggressive primary mucosal malignancy arising in the nasopharynx. It can directly spread anteriorly into the nasal cavity (AJCC, American Joint Committee on Cancer, 7th edition–stage T1) from which it can extend further into the PPF through the SPF (Fig. 5a and b). About 15 % of patients show involvement of the PPF at the time of diagnosis (AJCC stage T3) [14, 15]. From the PPF, the tumour commonly extends via the IOF into the orbit and via the PMF into the masticator space, both of which are categorized as AJCC stage T4 (Fig. 5c). PNS in NPC commonly occurs in a retrograde fashion, from the PPF to the middle cranial fossa along the V2 nerve, which is also considered stage T4 disease (Fig. 5d) [14–17].

Orbital invasion is seen in about 15 % of cases at the time of diagnosis. It is associated with sinister prognosis, with a 5-year survival rate of about 30 % [16–19].

The frequency of masticator space involvement is about 19.7 %. Its involvement is an independent prognostic factor for overall survival and local relapse-free survival of patients with NPC. These patients may require combined chemotherapy and RT as compared to RT alone in earlier stages of the disease. Also, higher doses of RT may be required [20].

Since RT remains the cornerstone of treatment in NPC, the identification of PNS is of paramount importance, as recurrence is guaranteed if it is missed in the RT field. Also, PNS and cranial nerve involvement are associated with poorer survival and poorer distant-metastases-free survival [9, 21].

#### Masticator space sarcomas

**Fig. 6:**
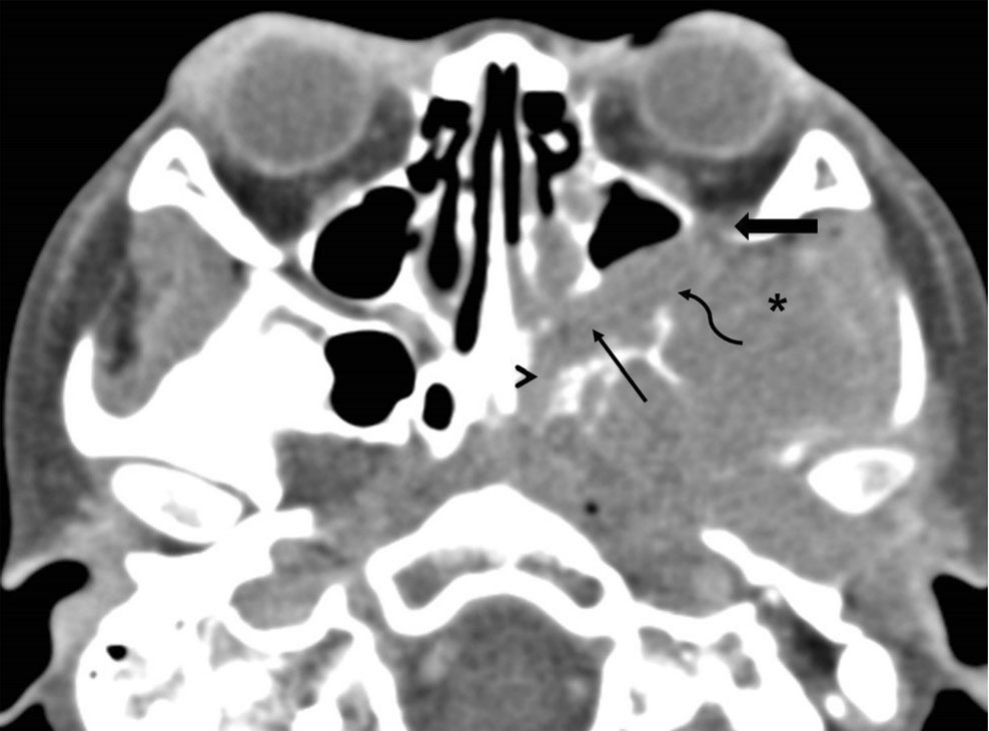
RMS of the left ITF in a 5-year-old boy. Axial CECT image shows an enhancing mass in the left ITF (*asterisk*) extending via the PMF (*curved arrow*) into the left PPF (*thin straight arrow*). The tumour extends via the IOF (*thick arrow*) into the left orbit. The left Vidian canal (*arrowhead*) is also involved

Mesenchymal tumours such as rhabdomyosarcoma (RMS), fibrosarcoma, etc. are common primary tumours of the masticator space/ITF. Sarcomas of the masticator space can directly extend medially via the PMF and infiltrate the PPF, which then acts as a highway for further intracranial spread of disease (Fig. 6) [22–25].

RMS of the PPF/ITF carries a poorer prognosis as compared to RMS arising in the nasal cavity, nasopharynx, and parapharyngeal region. In terms of management, tumours arising in PPF/ITF are usually considered unresectable and are treated with a combination of high-dose RT and chemotherapy. Despite the use of large fields and high-dose RT, local control remains a challenge and is the most frequent type of disease progression. Moreover, large RT fields and doses are associated with serious sequels like the impact on growth of facial bones in young children. Some authors suggest extensive surgery in combination with RT for improving local control in these tumours [26].

### PNS to the PPF

#### Cancers of the palate

**Fig. 7:**
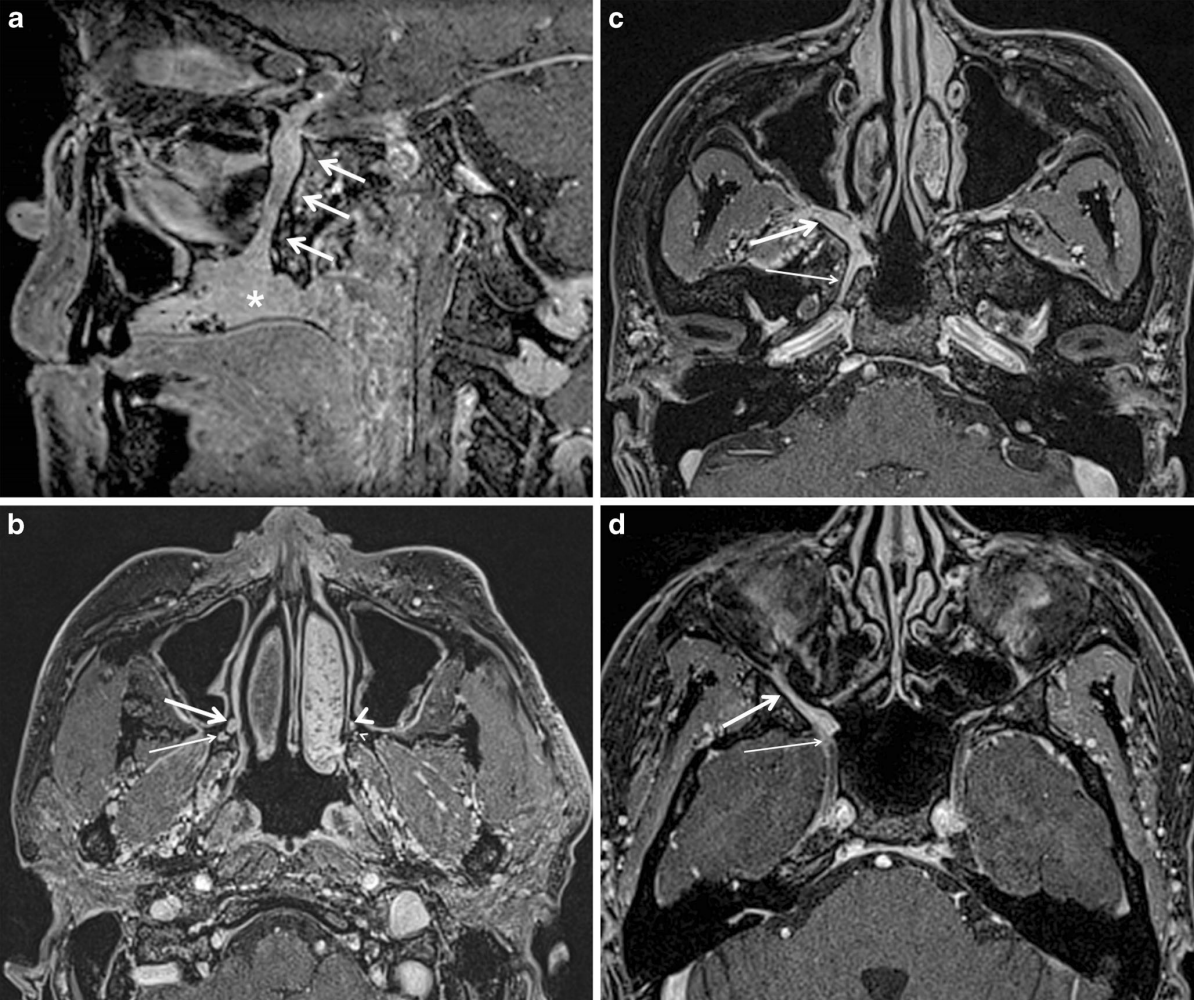
**a** ACC of the hard palate in a 33-year-old female patient. Sagittal contrast-enhanced FS T1W MR image shows an enhancing mass involving the hard palate (*asterisk*) with PNS along the right greater palatine nerve (*arrows*). **b** Axial contrast-enhanced FS T1W MR image in the same patient obtained at the level of the inferior apex of the PFF shows the enlarged, enhancing right greater palatine nerve (*thick arrow*) and lesser palatine nerve (*thin arrow*) due to PNS. Note the normal left greater palatine nerve (*thick arrow head*) and lesser palatine nerve (*thin arrow head*). **c** Axial contrast-enhanced FS T1W MR image in the same patient at a higher level shows disease spread from the right PPF (*thick arrow*) into the right Vidian canal (*thin arrow*). **d** Axial contrast-enhanced FS T1W MR image in the same patient obtained at the level of the FR (*thin arrow*). It shows a thickened and abnormally enhancing right ION (*thick arrow*) due to PNS

The palate has a high concentration of minor salivary glands and hence, tumours of minor salivary gland origin are very prevalent in the palate. Of these, the vast majority are adenoid cystic carcinomas (ACC). ACCs show a high propensity for PNS (almost 60 % cases). Squamous cell carcinomas (SCC), which arise in the hard palate, are also known to cause PNS [27]. The bulk of the neural supply to the palate is by the lesser and greater palatine nerves and hence, PNS from carcinomas of the palate commonly occurs in retrograde to the PPF via these two nerves. From the PPF, PNS further extends along V2 and the Vidian nerve into the middle cranial fossa and the cavernous sinuses (Fig. 7a, b, c and d). According to the 7th edition of UICC/International Union Against Cancer 2009, involvement of the PPF is not included in the T-staging of oropharyngeal (soft palate) or oral cavity (hard palate) cancers. Save for PNS along the ION in maxillary sinus tumours, PNS always indicates the highest T-stage in head and neck cancers according to the UICC staging system. It is considered an independent prognostic factor with a nearly three-fold increase in local recurrence and approximately 30 % decrease in 5-year survival rate [28, 29]. It portends the need for concurrent chemoradiotherapy, as opposed to surgical resection for smaller lesions (T1 or T2) restricted to the palate [30].

#### Cancers of the maxillary sinus

The maxillary sinus is most commonly affected by SCC and occasionally by ACC, both of which are known for PNS. The PPF may be directly involved by tumour spread through the posterior wall of the sinus or it may be involved by PNS via the posterior superior alveolar nerve(s) and/or ION (both being branches of V2) via the IOF [6, 10, 29, 31, 32]. Involvement of the PPF indicates stage 3 as per the UICC [28].

The presence of PNS is associated with increased risk of tumour recurrence, necessitating more aggressive treatment. Increased morbidity is a result of more extensive surgical resection, addition of adjuvant RT and/or widening of RT field [31, 32].

#### Cancers of the cheek

Cutaneous malignancies of the cheek like SCC and basal cell carcinoma can also extend perineurally to the PPF via branches of the ION. PNS occurs in approximately 2.5–5 % of primary cutaneous SCCs [33]. Accurate assessment of the presence and extent of PNS plays a pivotal role in the staging, management, and prognosis of patients with cutaneous SCC. In accordance with the AJCC, 7th edition, the presence of perineural invasion is considered a high-risk characteristic of T1 and T2 lesions, whilst the presence of perineural involvement of the skull base is categorized as T4. The involvement of larger calibre nerves (which are visible on imaging) is associated with an elevated risk of nodal metastasis and death. Along with the other risk factors (such as depth of invasion or degree of differentiation), PNS may require adjuvant RT along with surgical excision [33, 34].

#### Infective and inflammatory conditions

The PPF can be involved by the spread of invasive fungal sinusitis, bacterial sinusitis, and less often, in inflammatory conditions like inflammatory pseudotumour (IPT) [35–41].

#### Invasive fungal sinusitis

**Fig. 8:**
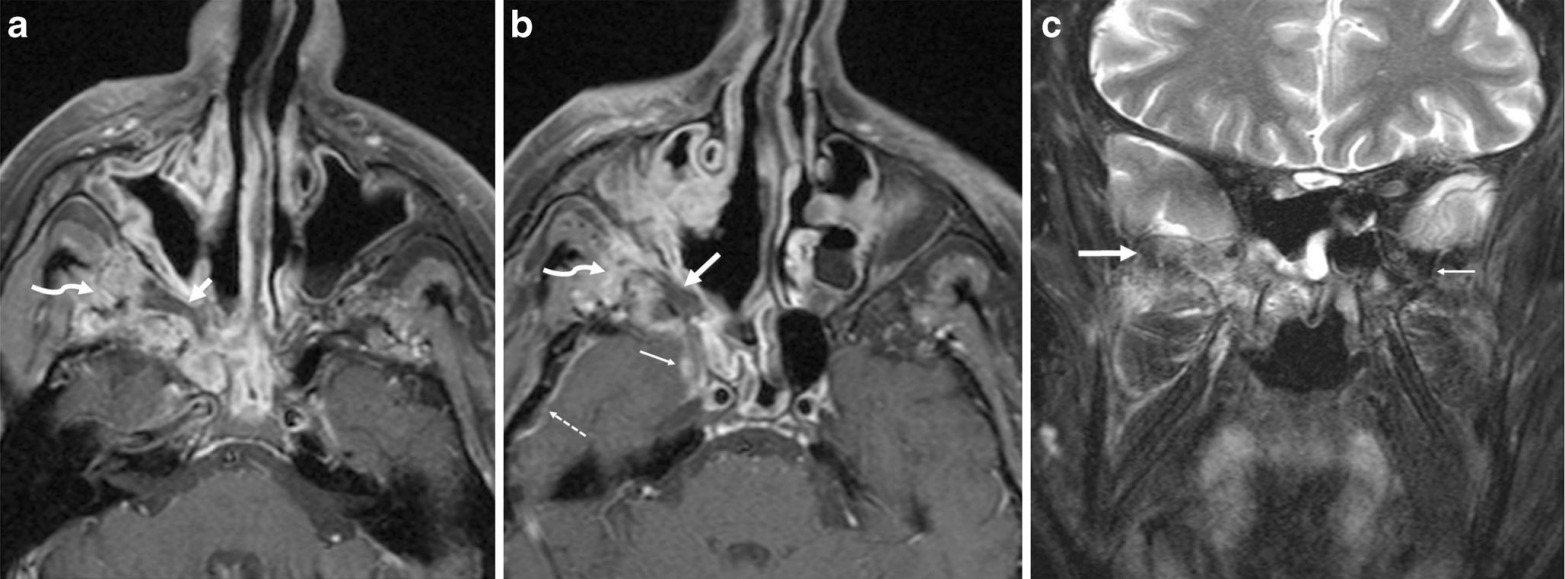
**a** Invasive fungal sinusitis caused by mucormycosis in a 54-year-old diabetic male. Axial contrast-enhanced FS T1W MR image shows right maxillary sinusitis with a formation of a rim-enhancing collection in the right PPF (*arrow*) in keeping with a fungal abscess (which was surgically drained later). Extension of the inflammatory changes into the right ITF (*curved arrow*) via the PMF. **b** Axial contrast-enhanced FS T1W MR image of the same patient shows involvement of the right V2 (*thin arrow*) and intracranial extension, as evidenced by the abnormal dural enhancement in the antero-lateral of the right middle cranial fossa (*dashed arrow*). The cranial aspect of the fungal abscess in the right PPF (*thick arrow*) with extension of the inflammation into the right ITF (*curved arrow*) and right sphenoid sinusitis. **c** Coronal inversion recovery MR image of the same patient shows marrow oedema of the right greater wing of the sphenoid bone (*thick arrow*) in keeping with fungal osteomyelitis. The left greater wing of the sphenoid bone (*thin arrow*) is normal

It commonly occurs in patients with immunocompromised states or uncontrolled diabetes (Fig. 8a, b and c). It is a progressive and often life-threatening disease, which often extends beyond the paranasal sinuses into the PPF, ITF, and intracranial cavity. Invasion of the PPF may occur either via direct erosion of the sinus walls of by perivascular infiltration. From the PPF, the disease shows easy access to the middle cranial fossa and cavernous sinus, leading to serious complications like cavernous sinus thrombosis, arterial mycotic aneurysms, infarcts, and abscesses. Extension into the PPF and ITF require rapid surgical debridement (which can be often very challenging in this area) in addition to aggressive antifungal therapy [35–38].

#### Bacterial sinusitis

**Fig. 9:**
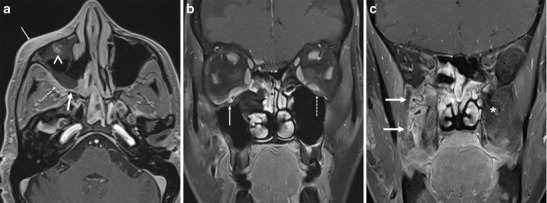
**a** Perineural spread of infection along the maxillary division (V2) of the right trigeminal nerve caused by E. coli infection of the right maxillary sinus in a 47-year-old female, who presented with fever and hypoesthesia along the right V2 dermatome. Axial contrast-enhanced FS T1W MR image shows right maxillary sinusitis involvement of the right PPF (*thick arrow*), PMF (*dotted arrow*), and the skin and subcutaneous tissue (*thin arrow*) overlying the right maxillary region. Thickening and abnormal enhancement of the right ION (*arrowhead*) due to perineural spread of infection is evident, thus resulting in hypoesthesia along the right V2 dermatome. **b** Coronal contrast-enhanced FS T1W MR image of the same patient shows asymmetrical thickening and abnormal enhancement of the right ION (*solid arrow*) secondary to perineural spread of infection from the PPF. Note the normal left ION (*dotted arrow*). **c** Coronal contrast-enhanced FS T1W MR image of the same patient shows abnormal enhancement with increased vascularization of the right PPF (*arrows*) due to its infective involvement by the right maxillary sinusitis. The contralateral PPF (*asterisk*) shows a normal appearance

Abscess formation in the PPF has been described secondary to dental/periodontal disease. Although rare, perineural spread of infection to the PPF can also be seen in bacterial sinusitis caused by E.coli, P. aeruginosa, or by streptococci. It should be suspected whenever patients with sinusitis present with symptoms related to any of the V2 branches (Fig. 9a, b and c) [39, 40].

#### IPT

**Fig. 10:**
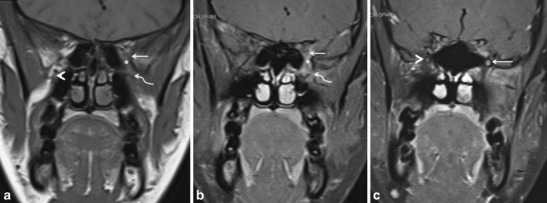
**a** A 35-year-old female presenting with painful opthalmoplegia of the left eye, diagnosed as IPT. Coronal T1-weighted MR image in the region of the orbital apex shows hypointense infiltrative soft tissue in the left orbital apex (*straight arrow*) extending inferiorly into the left PPF (*curved arrow*) via the left IOF (asterisk). Note the normal hyperintense fat within the right PPF (*arrowhead*). **b** Corresponding coronal contrast-enhanced FS T1W MR image in the same patient shows enhancing soft tissue in the left orbital apex (*straight arrow*) extending inferiorly into the left PPF (*curved arrow*) via the left IOF (*asterisk*). **c** Coronal contrast-enhanced FS TIW MR image in the same patient shows enhancement in the enlarged left foramen rotundum (*arrow*) in keeping with retrograde disease spread via the left V2 nerve. Note the normal right foramen rotundum (*arrowhead*)

IPT is a rare, biologically controversial entity in the head and neck that represents an idiopathic, non-granulomatous inflammation with myofibroblastic proliferation. IPT often behaves quite aggressively, resulting in wide-spread soft tissue involvement and bony destruction, which can make differentiation from a malignant lesion challenging. In the suprahyoid neck, IPT may affect the orbital contents, the paranasal sinuses and the masticator space. IPT is not associated with infection, neoplasm, or systemic disease; it has protean clinical manifestations depending on the type and location of the inflammation. Although IPTs most often arise in the orbit, extra-orbital extension may occasionally occur via the superior orbital fissure or IOF or via the optic canal. Involvement of the PPF (via the IOF). (Fig. 10a, b and c) is usually a mark of extensive disease and reflects a chronic process (present insidiously over months), which has been reported to have less favorable therapeutic outcomes as compared to the acute form [41–44].

#### Potential pitfalls in imaging of the PPF

Infective and inflammatory conditions in the PPF may mimic the perineural spread of malignancy. A schwannoma may also mimic PNS by causing thickening and enhancement of the affected nerve. Clinical correlation is essential to look for sinusitis/mucormycosis, etc. Image-guided fine-needle aspiration of abnormal soft tissue may provide further evaluation in selected cases [6, 29]. The PPF is commonly disrupted during surgical extirpation of large or posteriorly situated maxillary sinus tumours. After surgical violation, the PPF almost always appears abnormal on MRI with persistent abnormal soft tissue and often enhancement within. These expected imaging findings of scar tissue may be misdiagnosed as residual or recurrent disease. Obtaining an early post-operative baseline scan, and serial imaging for stability of findings, absence of new local mass lesion, and absence of new PNS are useful radiological tools in this scenario [7].

## Conclusion

The PPF is a ‘central station’ in the deep face where various neurovascular crossroads meet; it acts as a relay terminus for the spread of tumour, infection, and inflammation from one deep neck space to another. Hence, in pathologies of the adjacent spaces, it is necessary to review all the anatomical connections of the PPF to avoid missing out on subtle disease lurking in a blind spot. This pictorial essay serves to concisely review the anatomy of the PPF and to highlight diagnostic pearls and potential pitfalls while imaging PPF pathology.

## Abbreviations

PPF: Pterygopalatine fossa
PNS: Perineural tumour spread
PPG: Pterygopalatine ganglion
ITF: Infratemporal fossa
PMF: Pterygomaxillary fissure
IOF: Inferior orbital fissure
ION: Infraorbital nerve
SPF: Sphenopalatine foramen
NPC: Nasopharyngeal carcinoma
SCC: Squamous cell carcinoma
ACC: Adenoid cystic carcinoma
RMS: Rhabdomyosarcoma
IPT: Inflammatory pseudotumour
HRCT: High-resolution CT
NECT: Non-contrast-enhanced CT
CECT: Contrast-enhanced CT
CEMRI: Contrast-enhanced MR
FS: Fat-saturated
RT: Radiotherapy
AJCC TNM: American Joint Committee on Cancer (tumour, node, metastasis) classification
UICC: International Union Against Cancer
